# Binocular summation of chance decisions

**DOI:** 10.1038/srep16799

**Published:** 2015-11-18

**Authors:** Oren Yehezkel, Anna Sterkin, Dov Sagi, Uri Polat

**Affiliations:** 1 Goldschleger Eye Research Institute, Sackler Faculty of Medicine, Tel Aviv University, Sheba Medical Center, Tel Hashomer 52621, Israel; 2 The Weizmann Institute of Science, Rehovot 76100, Israel

## Abstract

Seeing with two eyes usually helps one respond faster. Here we show that with ambiguous stimuli, binocular viewing can paradoxically slow down reaction time. This is explained by the observers basing their decision on a noisy neuronal representation within the visual system, with the added noise breaking the symmetry between the two possible interpretations. Binocular integration improves the representation by reducing the noise, increasing ambiguity, and decision time. The neuronal Accumulator (Race) model is applied to quantify the underlying binocular integration. The model accounts for the distributions of reaction times, and predicts suboptimal integration between eyes. We conclude that under ambiguous stimulation neuronal noise within the visual system determines responses.

Commonly, performance benefits from binocular viewing as compared to monocular by about 20–40%^1^,^2^,^3^. Reaction time (RT) is also usually faster with two eyes compared to one^4^. Binocular integration has been widely studied in neurophysiology^5^,^6^ and psychophysics^1^ in a variety of tasks, with integration factors depending on tasks^7^ and stimulus contrast^8^,^9^. Here, we aimed at testing the advantage of binocular decisions under conditions of uncertainty. In the experiments, observers judged a dot matrix as perceptually grouped into “Columns” or “Rows” (Fig. 1; see Experimental procedures in Methods). Such a decision is affected by the ratio between vertical and horizontal dots spacings, with equal vertical and horizontal spacings allowing nothing but guessing. Human observers were tested under monocular and binocular viewing conditions using a randomized design with all different stimuli mixed in each block of trials (spacing ratios, monocular/binocular).

As expected^10^, the observers displayed a sharp transition between columns and rows perceptions (Fig. 2a): for larger spacing in the horizontal direction, dots grouped into columns, whereas for larger spacing in the vertical direction dots grouped into rows, with accuracy above 90%. However, in the ambiguous grouping condition, with equal spacing, the perception was at chance level (accuracy of ~50%). The results are similar for both monocular (Right eye or Left eye) and binocular viewing conditions.

However, reaction time measurements revealed a counterintuitive pattern of results. While RTs were similar for all unequal spacing displays, for both monocular and binocular trials (Fig. 2b), responses for ambiguous displays were significantly slower than any of the unequal spacing displays (p ≤ 0.013), as expected from the RT literature showing increased RT with decreasing performance level^11^. But, among ambiguous grouping trials, the binocular responses were unexpectedly slower by 89.4 ± 17.6 milliseconds (Mean ± SE, N = 21) compared to the average of the monocular trials (p = 5.6 × 10^−5^); 2/3 of the subjects had slower binocular responses, but no one had faster binocular RTs. One possible explanation is that observers base their decision on a noisy neuronal representation of the stimulus, with the noise level serving as a “signal”. Binocular integration is expected to reduce the noise and thus the strength of the signal used for decision, leading to slower RTs.

Temporal aspects of decision making are well captured by a family of “Accumulator” or “Race” models^11^,^12^,^13^,^14^,^15^ (see RT distribution and Accumulator (Race) model fit in Methods). According to this theory, evidence accumulates with time separately for each possible decision (in our case Columns and Rows). The winning accumulator is the one that reaches first a threshold level, and thus determines the decision. The critical parameter here is the accumulation rate, which sets the speed of the decision process. We suggest that in ambiguous grouping conditions, the evidence accumulated is determined by neuronal noise added to the otherwise equal stimulus-evoked responses. This noise is reduced by binocular integration, thus making the neuronal responses supporting the two decision outcomes more similar.

To test the applicability of the accumulator model to our data, we fitted model predictions for RT distributions to our experimental results in the ambiguous conditions (the non-ambiguous conditions produced close to perfect discriminations thus are not informative). The model assumes that each accumulator is governed by a diffusion-like process to a single boundary, where the accumulated evidence is incremented on each time step by an amount representing the momentary evidence (μ_h_, μ_v_ corresponding to the horizontal and the vertical accumulators), added with Gaussian noise (σ = 1), and terminated when the first accumulator reaches the decision boundary (λ). Fig. 3a presents the outcome of these fits, showing an excellent match between model and data. We assumed μ_h_ = μ_v_ = μ for ambiguous grouping, as expected from balanced stimuli, and since all experimental conditions were randomly mixed in one experimental session, the decision boundary (λ) was kept constant across conditions (see RT distribution and Accumulator (Race) model fit in Methods). The fitted parameters show μ_R_ = 0.057, μ_L_ = 0.059, and μ_B_ = 0.048, for the right-eye, left-eye, and two-eyes conditions respectfully (Fig. 3b). Thus, while the monocular accumulation rates are very similar, the binocular rate is 21% lower (95% confidence range of 13–31%), which we interpret as 21% noise reduction due to information averaging between the eyes. This value broadly agrees with several published values of binocular summation^9^,^16^.

Our results suggest that binocular integration leads to noise-reduction, in agreement with previous results showing improved detection thresholds with two eyes^1^,^2^,^3^. A noisy system optimally integrating two noisy sources would show 41% gain (√2), thus the summation factor found here is sub-optimal, possibly pointing to a correlation between the two noise sources. It is noteworthy that the current accumulator model was only applied here to demonstrate the very ability to quantify the summation factor. Other models may provide a theoretical support for the predicted inter-ocular integration, such as Bayesian or sensory recalibration models^13^,^17^,^18^,^19^,^20^,^21^,^22^, but these models are not directly applicable to RT.

Using a very simple perceptual grouping task we showed that observers’ guessing behavior can be explained by them accessing neuronal noise within the visual system. This proposal, combined with the Race model of human decision, explains the paradoxical slow-down of reaction times for decisions based on binocular viewing relative to monocular viewing.

## Methods

### Experimental procedures

#### Observers

Twenty one observers participated in the experiments (ages ranged from 17 to 35), with normal or corrected-to-normal visual acuity, unaware of the purpose of the study. Each observer signed an informed consent form approved by the local Institutional Review Board of Sheba Medical Center.

#### Apparatus

The experiments were controlled by a PC and the stimuli were displayed as a gray-level modulation on a Philips 107P color monitor, 100 Hz refresh rate. The mean display luminance was 20 cd/m^2^ in an otherwise dark environment. Screen resolution was 1024 × 768 pixels; gamma correction was applied. The stimuli were viewed from a distance of 150 cm.

#### Stimuli

**Figure 1:**
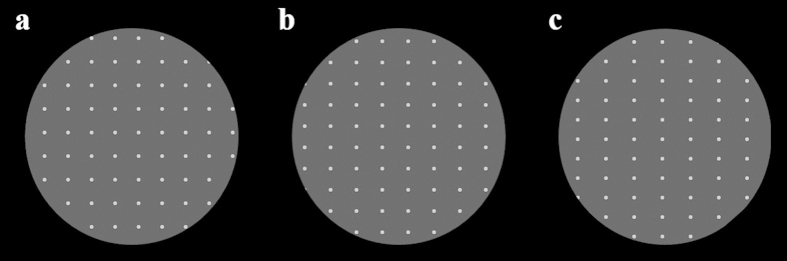
Proximity grouping. A forced-choice procedure was used, in which subjects had to decide whether they perceive brief matrices of white dots on black background as rows or columns. The proximity grouping performance was assessed at different inter-dot spacing ratios along the horizontal vs. the vertical axes. The ratio could be (**a**) equal, i.e., 0 change in any direction, producing an ambiguous grouping, with an equal probability of perceiving rows or columns, or (**b**) changed by 10% and (**c**) by 20% in each direction. Trials were presented, either to both eyes (binocularly), or only to one (monocularly), while the other exposed to background luminance, randomized, using stereo goggles to keep the observer unaware of the trial type. To avoid the global matrix shape cues, dot matrices were presented via a round window with a 5-cm radius occupying 3.8 degrees of the visual field. Each trial consisted of a binocular central fixation mark, followed by an observer-triggered 80-millisecond dot matrix. Observers received no feedback on their responses.

A matrix of white dots was presented on a gray background, each dot occupying 3 × 3 pixels (Fig. 1). Dot size was 2.5 min of arc. Dots intensity was 45 cd/m^2^. The experimental variable was the ratio of vertical to horizontal spacing between the centers of the dots^23^. According to the Gestalt law of proximity, when a dot matrix is presented with a different gap size in the horizontal or vertical direction, the more proximal dots tend to form perceptual groups, in such a way that the matrix can be perceived as columns, rows, or as an ambiguous matrix pattern. The distance between the dots was changed along both the vertical and the horizontal direction; the 5 spacing ratios tested were 20 and 10 percent in each direction and equal spacing (i.e., 0 change in either direction). The spacing between the dots within the dot-array was 33.75 min of arc for the equal spacing. Stimuli were presented, in a random order either to both eyes, or only to one while the other exposed to background luminance, using stereo goggles (Crystal eyes 3, StereoGraphycs). Observers were unaware of the eyes targeted on each trial.

#### Visual task

The task was to report the perceptual organization of the display as horizontal Rows or vertical Columns, without feedback, using a forced-choice paradigm, by pressing a computer mouse button immediately after making their decision using the dominant hand. Each trial was preceded by a binocular fixation mark at the center of the display until the observer signaled their readiness using the computer mouse. Fixation mark disappeared and after 300 msec a stimulus was presented for 80 msec. The matrix had a rectangular shape in most cases, due to the unequal spacing between the rows and columns, which may have interfered with the observers’ judgment. To avoid such interference, the screen was covered with a round window, so that the global form of the stimulus was circular across all experiments, occupying 3.8 deg of the visual field.

**Figure 2:**
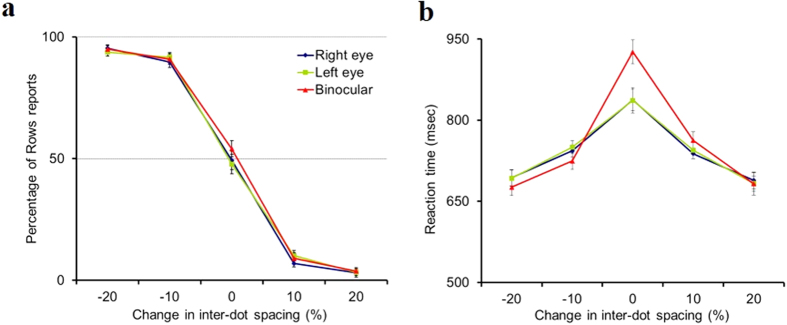
Slower binocular responses during ambiguous grouping. The X axis represents changes in inter-dot spacing in the horizontal (positive) and the vertical (negative) directions, as the percent of the spacing used for equally spaced displays. (**a**) The Y axis represents the percentage of horizontal judgments (Rows), and (**b**) the mean RT (N = 21; 40 trials per datum point/observer). Regarding grouping reports (**a**), for the 5 spacing ratios, observers showed no significant trial type effect (F_2,19_ = 1.6, P = 0.21) and a significant spacing ratio effect (F_4,17_ = 720.5, P < 0.001), with no interaction. For spacing ratio of 1, observers showed no significant trial type effect (F_2,19_ = 2.135, P = 0.136). Regarding RT (**b**), for the 5 spacing ratios, observers showed no significant trial type effect (F_2,19_ = 2.9, P = 0.065) and a significant spacing ratio effect (F_4,17_ = 28.7, P < 0.001), with interaction (P = 0.008). For spacing ratio of 1, observers showed a significant trial type effect (F_2,19_ = 11.5, P < 0.001). All pairwise comparisons used paired t-test. Non-significant effects indicate P > 0.05. Error brackets are SE.

Data were collected in sessions of at least 600 trials per observer (all but 5, for which 300 trials were collected) that lasted ~15 minutes (the actual number of trials was larger than 600 in order to keep block randomization to the end of the session). Within each session, trials of all 15 conditions (5 distance ratios and 3 eye combinations) were randomly mixed. Observers’ performance, quantified as the percentage of trials reported as Rows (horizontal), was calculated for each spacing ratio. Reaction times were measured as the time from stimulus onset to response. Group results (Fig. 2) are averages of individual observers’ means.

#### Statistical analysis

ANOVA results are reported with Greenhouse-Geisser Epsilon adjustment. Paired two-tailed t-test was used for pairwise comparisons. ANOVA included 3 trial types (right-eye, left-eye and binocular) and 5 spacing ratios. The significance of the bias for ambiguous grouping was calculated using a one-sample t-test compared to the predicted value of 50. The bias was 0.67 ± 3.75%, 2.24 ± 3.9%, −4.1 ± 3.3% (mean ± SE, N = 21) for the trials viewed with the right eye, the left eye or binocularly, respectively (p = 0.86, 0.57 and 0.23 for the trials viewed with the right eye, the left eye or binocularly, respectively).

### RT distribution and Accumulator (Race) model fit

To model reaction time distributions in our 2-alternative forced choice experimental paradigm, we assume a “race” between two independent accumulators (i.e. one for Rows and one for Columns), with a response produced according to the first one to reach the boundary^11^. Each accumulator is modeled by a single-boundary diffusion whose completion time (i.e., time to boundary) is Wald distributed. In the ambiguous condition, both accumulators received equal supporting evidence, and hence equal drift rate, thus the decisions were driven by the added noise. The decision time probability density function for such a race model can be computed as the product of two terms: the first describing the probability that one accouter reached its boundary at time t, and the second that the other accumulator is still running.


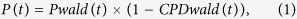


where P*wald*(t) is given by:


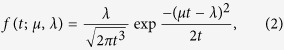


for reaction times t > 0, where μ > 0 is the drift rate and λ > 0 is the decision boundary, with CPD*wald*(t), the cumulative Wald distribution, given by:


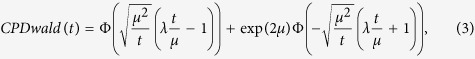


where Φ is the standard normal (Gaussian) distribution c.d.f.

The distribution of the observers’ reaction times were fitted to eq. (1) using a maximum likelihood estimation algorithm (Matlab©, mle()). Of interest here is the conditions with equal vertical and horizontal spacing, producing chance-level performance, modelled using two accumulators with equal drift rates, assuming the same decision boundary for all conditions (left-eye, right-eye, binocular). In the fitting process we included two additional parameters, assumed to be equal in all conditions: the decision boundary (λ = 40), and the minimum RT accounting for visual transmission time and motor reaction (often termed “non-decision time”, rt_0_ = 300 milliseconds) subtracted from the measured RT (these two parameters were estimated separately for each condition, found to be very similar thus the averaged estimates were used as constants while refitting the drift-rate parameters). For the analysis we ignored the first 5 trials of each session, and RTs within the lower and upper 2.5% percentiles^24^. Observers having a strong bias in the ambiguous conditions (N = 5), deviating significantly from the expected chance performance, were not included in this analysis. Their bias was strong and consistent across viewing conditions (right-eye/left-eye/binocular) and is thus suspected to reflect an automatic response in case of high uncertainty (i.e., “I don’t know so I press right key”) and their distributions may differ. Therefore, in order to be sure that chance performance is at the individual-observer level, not a result of averaging between strongly biased observers of opposite biases, for the sake of model’s clear application to unbiased chance responses, observers with these biases were excluded^25^. To identify strongly biased observers, response distributions of the individual observers were compared to a binomial process over the same number of trials. The critical bias was set to 4SE_binom_, with SE_binom_ calculated according to the expected SE from the binomial processes. Overall, the RT distribution analysis was based on 2030 trials. The goodness of fit was assessed using the Chi-squared test, performed on 5 equally spaced bins to meet the minimal count requirement, according to the 0.2, 0.4, 0.6 and 0.8 quantiles^24^. For the resulting p-value of 0.27 we do not reject the null hypothesis, indicating a good fit.

## Additional Information

**How to cite this article**: Yehezkel, O. *et al.* Binocular summation of chance decisions. *Sci. Rep.* **5**, 16799; doi: 10.1038/srep16799 (2015).

## Figures and Tables

**Figure 3:**
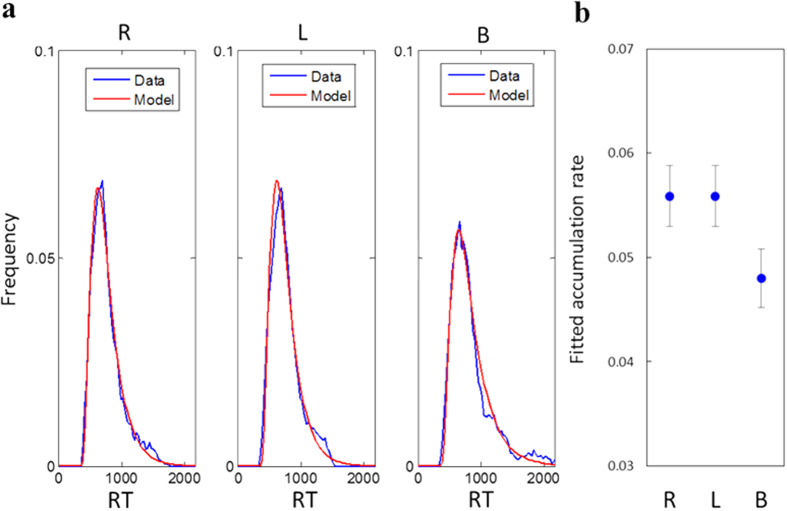
(**a**) RT distributions, data (N = 16 observers) and model (see Methods), shown for the right eye (R), left eye (L), and for the two eyes (binocular, B) viewing conditions. (**b**) Accumulation rates obtained from fitting model to RT data from ambiguous grouping trials, with error bars showing 95% confidence intervals.
